# Task-switching Cost and Intrinsic Functional Connectivity in the Human Brain: Toward Understanding Individual Differences in Cognitive Flexibility

**DOI:** 10.1371/journal.pone.0145826

**Published:** 2015-12-30

**Authors:** Shouhang Yin, Ting Wang, Weigang Pan, Yijun Liu, Antao Chen

**Affiliations:** Key laboratory of Cognition and Personality of Ministry of Education, Faculty of Psychology, Southwest University, Chongqing, China; Institute of Psychology, Chinese Academy of Sciences, CHINA

## Abstract

The human ability to flexibly alternate between tasks (i.e., task-switching) represents a critical component of cognitive control. Many functional magnetic resonance imaging (fMRI) studies have explored the neural basis of the task-switching. However, no study to date has examined how individual differences in intrinsic functional architecture of the human brain are related to that of the task-switching. In the present study, we took 11 task-switching relevant areas from a meta-analysis study as the regions of interests (ROIs) and estimated their intrinsic functional connectivity (iFC) with the whole brain. This procedure was repeated for 32 healthy adults based upon their fMRI scans during resting-state (rfMRI) to investigate the correlations between switching cost and the iFC strength across these participants. This analysis found that switch cost was negatively correlated with a set of iFC involved ROIs including left inferior frontal junction, bilateral superior posterior parietal cortex, left precuneus, bilateral inferior parietal lobule, right middle frontal gyrus and bilateral middle occipital gyrus. These connectivity profiles represent an intrinsic functional architecture of task-switching where the left inferior frontal junction plays a hub role in this brain-behavior association. These findings are highly reproducible in another validation independent sample and provide a novel perspective for understanding the neural basis of individual differences in task-switching behaviors reflected in the intrinsic architecture of the human brain.

## Introduction

The constantly changing environment in our life demands cognitive control system to flexibly coordinate thoughts and behaviors in order to accomplish internal goals. A basic component of cognitive control involves the ability to reconfigure task-sets in a flexible manner in order to meet shifting demands [1]. The common way to investigate this cognitive ability is through the use of the task-switching paradigm [2]. In task-switching paradigms, participants are required to perform two or more different tasks in a rapidly intermixed manner [3, 4], with an advance cue typically indicating the task to be performed on the upcoming trial [5]. There is performance decrement in task-switching relative to task repetitions, which is termed switch cost [1]. Functional magnetic resonance imaging (fMRI) studies of task-switching have identified a distributed and often left-lateralized network of frontoparietal brain areas that contribute to task-switching, including dorsolateral prefrontal cortex (DLPFC), ventrolateral prefrontal cortex (VLPFC), frontopolar cortex (FPC), pre-supplementary motor area (pre-SMA) and posterior parietal cortex (PPC) [6–10]. Furthermore, a brain region termed as left inferior frontal junction (IFJ), which is anatomically located at the junction of the inferior frontal sulcus and the inferior precentral sulcus, served as a crucial role in task-switching and set shifting [11]. However, previous neuroimaging studies have primarily tended to describe what role these brain regions play in task-switching, and little is known about how these brain regions are organized to form an intrinsic network for the performance of switching between different tasks.

Recently, some substantial advances in understanding human brain organization have emerged from a relatively unusual approach: the correlated patterns in spontaneous activity, in the ‘resting’ brain (for a comprehensive review, see [12]). Specially, by placing seed regions in brain areas, many functional networks have been shown to be coherent in their spontaneous activity, including the somatomotor [13, 14], visual [15, 16], default mode [17, 18], language [15, 19], memory [20, 21] and dorsal and ventral attention networks [22]. The intrinsic functional connectivity (iFC) analysis provides a new insight for us to understand the intrinsic functional architecture in human brain and the relationship between the iFC and the actual behavior. Hampson et al. (2006) reported that the posterior cingulate cortex (PCC) and ventral anterior cingulate cortex (vACC) were functionally connected during a working memory task and at rest, and the performance was positively correlated with the iFC strength during both the working memory task and rest [23]. Song et al. (2008) found that iFC of DLPFC were correlated with intelligence scores in multiple brain regions [24]. The iFC is changeable by various training processes such as a perceptual learning [25] and predictable with cognitive performance [26], behavioral deficits [27], and learning outcomes [28, 29]. Examination of the relationship between the spontaneous brain activity measured with iFC and the individual differences in human behaviors can reveal the intrinsic functional architecture for understanding the neural basis of the individual differences in specific behaviors.

In the current study, we explored the relationship between the iFC and individual differences in task-switching. Participants were recruited to perform a resting-state fMRI scan, and after the rfMRI scan, outside of the scanner, they achieved a classic switching task [30] to evaluate the magnitude of the performance on switching between different response rules or opposing stimulus-response (S-R) mappings. The switch cost was defined as the difference in response time (RT) between the switch and the repeat trials when alternating between two tasks (switch cost = switch trial RT—repetition trial RT) [4]. Specifically, 11 cortical seed regions of interest (ROI) adopted from a meta-analysis study of task-switching with fMRI [31] were used in the iFC analyses. These meta-seeds are highly reliable across the previous studies and are the most representative regions involved in task-switching performance [32]. To ensure the reliability of our findings [33], we incorporated an independent sample to verify the results.

## Materials and Methods

### Participants

The procedure of this study was approved by the Ethics Committee of the Southwest University. Sample 1 consisted of 32 right-handed undergraduates (21 females) aged 19–25 years (mean age = 22.3, SD = 1.67) from Southwest University in China. Sample 2 consisted of 28 right-handed undergraduates (12 females) aged 18–24 years (mean age = 20.5, SD = 1.4) from the same population. Five additional participants were enrolled in these two samples, but their data were excluded from further analysis because of excessive head motion (which exceeded 2.0 mm in transition or 2.0 degrees in rotation). All participants had no neurological disease and normal or corrected-to-normal visual acuity. All participants gave written informed consents before the brain imaging session and were paid for participating.

### Behavioral tasks

**Fig 1:**
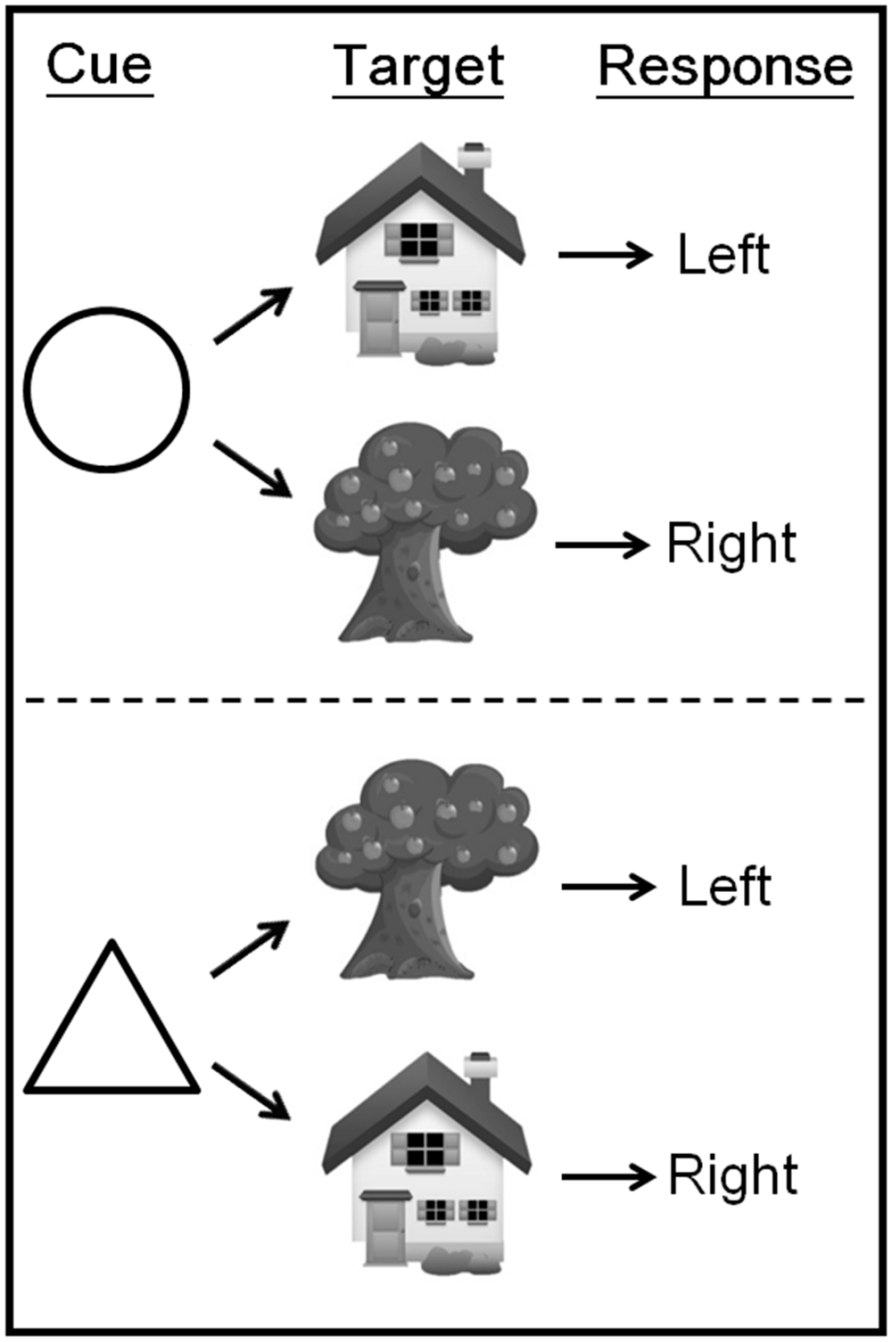
Display of rule types. During scanning, participants viewed an instructional cue for 1 s. After a 0.5 s delay, the target stimulus was presented for 2.5 s. The target required a left- or right-button response, depending on the relevant S–R mapping learned before scanning.

Participants performed the behavioral tasks soon after the rfMRI scanning, which was described with details in following subsection. They need to learn how to associate each of two visual cues with a set of S-R mappings (Fig 1). The task involved a visual cue that instructed the participants which rule to use, followed by a target stimulus that required a left- or right-button response. The cue could be a circle or a triangle. A house or a tree could follow the circle cue, and participants were instructed to respond with a left-button press to the house and with a right-button press to the tree. A house or a tree could also follow the triangle cue, but for this cue the S-R mapping was reversed: the house was associated with a right-button response and the tree with a left-button response. The response key sequences were counterbalanced across participants.

Each trial was 5.5 s long, and had the following structure: a fixation cross was presented for 1 s, followed by a 1 s cue (circle, triangle), and then by the target after a 0.5 s delay (blank screen). The presentation of the target was response-terminated, but responses had to be given within 2.5 s. The trial would terminate when the response was given within the 2.5 s response time window. In practice, participants performed twelve single rule tasks, and several mixed tasks (two rules presented with pseudo-random) with feedback and didn’t stop until they answered properly for eight times in a row. In this session, there was a right or wrong feedback following the participants’ response. Participants then performed ten real blocks (17 trials; switching: repetition = 1:1) with no feedback. The first two blocks were designed to make participants get used to real tasks and were excluded in further data analysis. Participants were instructed to press the responding key as quickly and as accurately as possible.

It was reported that if the cue-target interval (CTI) was long enough for participants to prepare for the task, only residual switch cost can be measured [34]. However, how long the CTI was enough for the full preparation is specific for a task. Accordingly, we conducted a behavioral experiment to resolve this problem for our task design. Additional twenty-two participants took part in this experiment, and the procedure was similar as mentioned above. The only difference is that the CTI was changed between blocks (116, 1516, and 2916 ms; six blocks) and was kept constant within a block. The results clearly showed that the switch cost was not only measuring the residual of switch costs when the CTI was around 1500 ms. Compared with the 2916 ms, when the CTI was 1516 ms, the switch cost was remarkably greater [70 ms vs 19 ms; *t(21)* = 2.99; *p* < 0.01]. This indicates that some participants failed to take full advantage of the cue-target interval to prepare the task.

### MRI Image acquisition

MRI data were obtained using a 3.0 Tesla Siemens scanner (Siemens Magnetom Trio TIM, Erlangen, Germany). Each participant in Sample 1 underwent an rfMRI scan lasting 7 minutes and 45 seconds. During this time, participants were instructed to keep their eyes staring at the fixation “+” but not fall asleep, relax and remain motionless. The rfMRI scan comprised 184 contiguous whole-brain functional volumes with an echo planar imaging (EPI) sequence [TR = 2500 ms; TE = 30 ms; flip angle = 80°, matrix = 72 × 72, 38 inter-leaved 3 mm-thick slices, resolution = 3 × 3 mm^2^, slice skip = 0.33 mm]. A high-resolution T1-weighted anatomical image was also acquired using a magnetization prepared gradient echo (MPRAGE) sequence [TR = 2600 ms; TE = 3.02 ms; TI = 900 ms; flip angle = 8°; 176 slices]. In Sample 2, the rfMRI scan contained 240 contiguous whole-brain functional volumes in 8 minutes [TR = 2000 ms; TE = 30 ms; flip angle = 90°, matrix = 64 × 64, 33 inter-leaved 3 mm-thick slices, resolution = 3.1 × 3.1 mm^2^, slice skip = 0.6 mm]. A high-resolution T1-weighted anatomical image was also acquired [TR = 1900 ms; TE = 2.52 ms; TI = 900 ms; flip angle = 9°; 176 slices].

### Seed regions of interest

**Fig 2:**
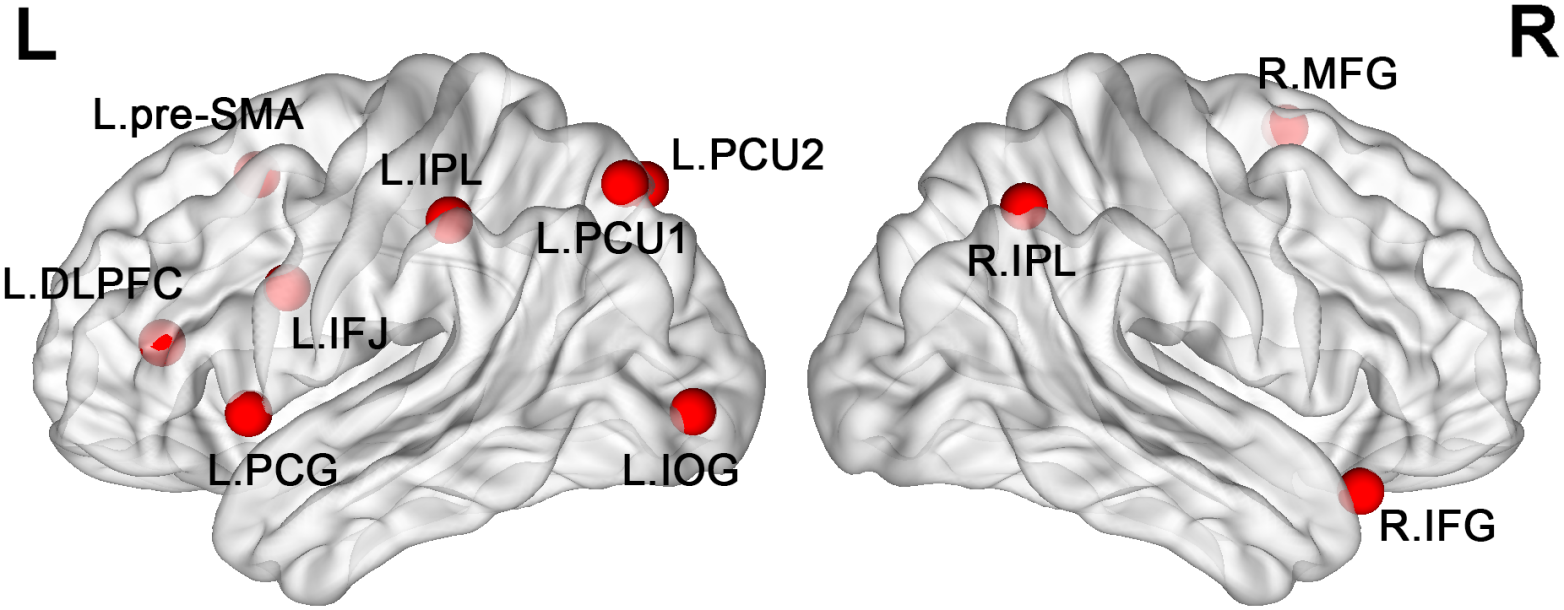
Seed regions-of-interest (ROIs). Eleven seed regions were selected from a meta-analysis study of task-switching. Note: L = left; R = right; pre-SMA = pre-supplementary motor area; IFJ = inferior frontal junction; PCG = precentral gyrus; DLPFC = dorsal lateral prefrontal cortex; IPL = inferior parietal lobule; PCU = Precuneus; IOG = inferior occipital gyrus; IFG = inferior frontal gyrus.

We determined seed ROIs based upon eleven cortical seed regions reported in a previous meta-analysis on task-switching [31]. For each seed, we converted the reported Talairach coordinates into the Montreal Neurological Institute (MNI) coordinates in the Resting-State fMRI Data Analysis Toolkit (REST) [35], and then created a spherical ROI centering at the MNI coordinates with a radius of 6 mm (for the exact locations of these ROIs, see Fig 2 and Table 1).

**Table 1:** Eleven Meta Seed Regions of Interest (ROIs).

Seed regions of interest	MNI coordinates			BA
	x	y	z	
Left pre-supplementary motor area (L.pre-SMA)	-7	17	48	6
Left inferior frontal junction (L.IFJ)	-44	4	29	6
Left precentral gyrus (L.PCG)	-51	12	0	44
Left dorsal lateral prefrontal cortex (L.DLPFC)	-36	35	13	46
Left Precuneus (L. PCU1)	-22	-73	52	7
Left inferior parietal lobule (L.IPL)	-46	-35	47	40
Left Precuneus (L. PCU2)	0	-75	48	7
Left inferior occipital gyrus (L.IOG)	-33	-91	0	18
Right pre-supplementary motor area (R.pre-SMA)	4	10	59	6
Right inferior frontal gyrus (R.IFG)	44	21	-20	47
Right inferior parietal lobule (R.IPL)	41	-58	50	7

BA = Brodmann area.

### Image preprocessing

Preprocessing was performed using Data Processing Assistant for Resting-State fMRI [36], which was built on Statistical Parametric Mapping software (SPM8; http://www.fil.ion.ucl.ac.uk/spm). The first 10 rfMRI volumes were discarded for signal equilibrium and adaptation of the participants to the scanning noise. Slice timing and head motion correction were then performed, and a mean functional image was obtained for each participant. To spatially normalize functional images, each participant’s structural brain image was co-registered to the mean functional image and was subsequently segmented. The registration parameters obtained in the segmentation were used to transform each participant’s rfMRI image into the MNI space (resampling voxel-size: 3 × 3 × 3 mm^3^). Spatial smoothing (6 mm FWHM Gaussian kernel) was conducted to reduce spatial noise, and the linear trend was removed from the preprocessed rfMRI time series. Finally, a band-pass filter (0.01–0.08 Hz) was applied to reduce low-frequency drift and high-frequency noise.

### Seed-based iFC computation and iFC-behavior correlation analyses

Before conducting functional connectivity analyses, we first regressed out white matter, cerebrospinal signals and head motion parameters estimated by Friston 24-parameter model [37] for head motion correction, which was considered better for individual-level correction [38], from the individual preprocessed rfMRI time series. We then conducted seed-based iFC computation and multiple comparison correction within a grey matter (GM) mask, which included 42,520 voxels without cerebellar.

We first calculated the seed × seed partial correlation matrix of each participant. The partial correlation coefficients (eliminating the contribution of another 9 ROIs) of pairwise regions of 11 ROIs were calculated in both the two samples. Then, the relationship between switch cost and the partial correlation matrix of each participant were examined. However, no iFC-behavior correlations were significant after the multiple comparison correction. Since the peak coordinates of some regions with wide range (e.g. PPC) would vary in different samples, the direct calculation of the iFCs between the peak coordinates of each ROI could be inaccurate. The coordinates of two regions showing switch cost relevant iFCs in the present sample would be somewhat different from the priori peak coordinates of the two regions. Hence, to further explore the intrinsic functional architecture of task-switching, we calculated the iFC profiles of each ROI, and then examined the iFC-behavior correlations. We expected that these analyses could identify the switch cost relevant iFCs which involved the eleven switch-related regions.

The iFC analysis was performed using the REST software [35]. Specifically, for each participant, mean time series of each ROI was calculated and then correlated with time series of all other voxels in the whole brain. The correlation coefficients were converted into Fisher z-values to generate a z-functional connectivity (zFC) map for further statistical analysis. This led to eleven zFC maps for each participant.

We conducted iFC-behavior correlation analyses to identify those iFCs whose strength might predict behavioral performance of task-switching. The computation was implemented by using the ‘REST Correlation Analysis’ command in the REST software, which calculated the Pearson’s correlation coefficient between the zFC strength and switch cost for each voxel. Of note, participants’ age, gender, mean reaction time and mean accuracy were controlled for this group-level analysis. These results were then corrected for multiple comparisons with *AlphaSim* (originally in AFNI software and re-implemented in REST, see more details of the *AlphaSim* command at http://afni.nih.gov/afni/docpdf/AlphaSim.pdf). The parameters were as follows: individual voxel p-value = 0.01, 1000 simulations, two-sided, FWHM estimated by 6 mm FWHM, cluster connection radius = 5 mm (edge connected), with the GM mask. According to the simulations, a corrected significance level of p < 0.01 could be achieved with a spatial cluster threshold of 55 voxels.

Several iFCs that focused on the left IFJ and PPC were revealed by these analyses. To further find the specific connectivity, we calculated the whole brain partial correlation mappings of these two regions, and then examined the partial iFC-behavior correlations. This analysis was conducted in all the individuals from the two samples. A multiple linear regression model was built where the time series of one ROI was regarded as the dependent variables, and another ten ROIs’ signals were regarded as explanatory variables. We saved the residual as the signals of one ROI, which were correlated with time series of all other voxels in the whole brain. The subsequent analyses were same as the above-mentioned iFC-behavior correlation analyses.

### Test-validation procedure

To test the validity and reproducibility of the findings, we collected behavioral and rfMRI data from another set of 28 independent participants from the same population. The zFC maps for these 28 subjects were generated using the same procedure as in Sample 1. We then extracted mean zFC from the ROIs identified by the brain-behavior correlation analysis in Sample 1 for the 28 participants. We performed a ROI-wise (in contrast to the voxel-wise in Sample 1) correlation analysis between the zFC strength and switch cost.

Furthermore, to eliminate the influence of head movements and the global signal for rfMRI data, we conducted several tests including different preprocessing strategies to ensure the reproducibility of these findings. The below mentioned methods were used for preprocessing the rfMRI data in Sample 1, and the iFC-behavior correlation analysis was also conducted in Sample 1. First, we use the traditional rigid-body 6-parameter model to replace Friston 24-parameter model: regressing out 6 head motion parameters (i.e., three translations and three rotations). Second, we added scrubbing data into the initial Friston 24-parameter model (spike regression): identifying “bad” time points using a threshold of frame-wise displacement (FD) > 0.2 mm as well as 1-back and 2-forward neighbors as done in Power et al. (2013) [39], and modeling each “bad” time point as a separate regressor in the regression models. Furthermore, we added the global signal as a separate regressor into the initial Friston 24-parameter model to remove the effect of the global signal on the findings. We only tested those results, which were reproducible in Sample 2.

## Results

### Behavioral results

**Fig 3:**
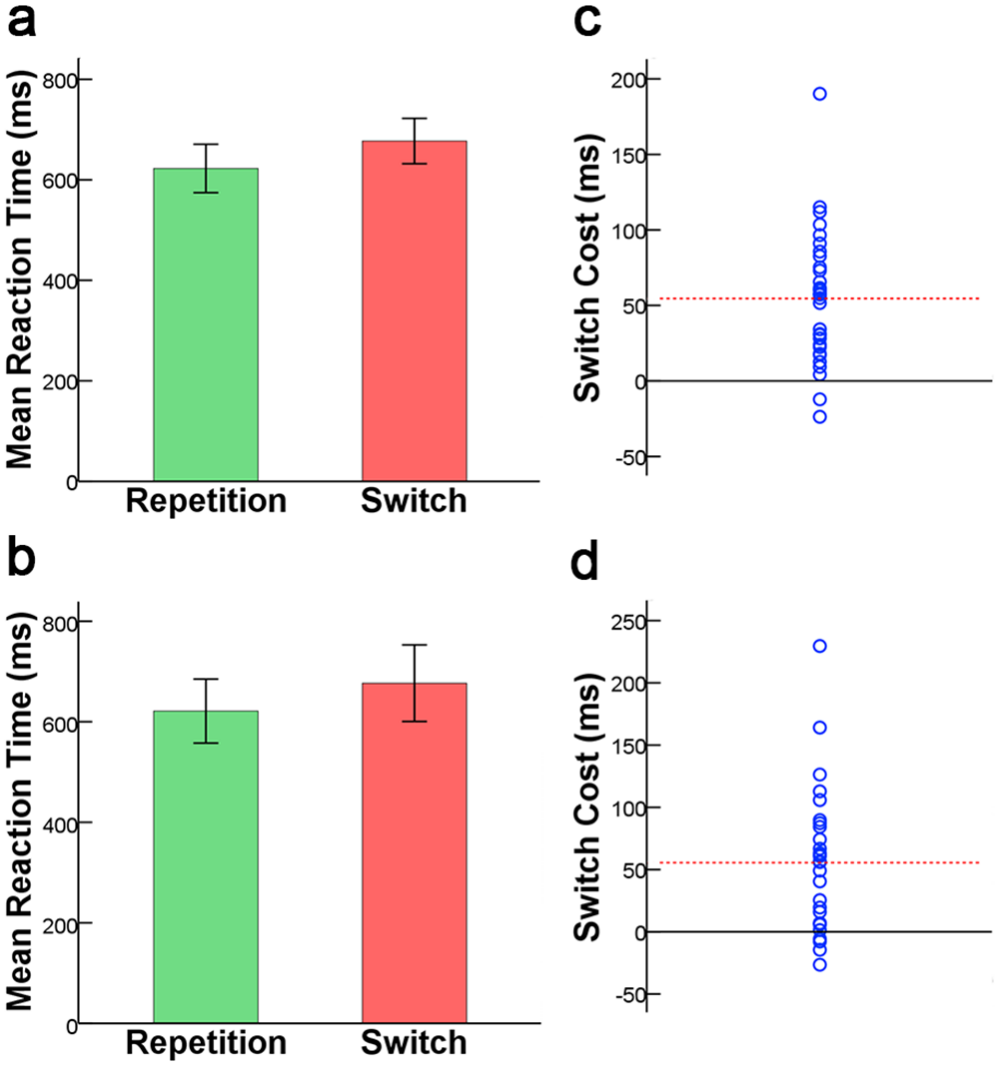
RT results and switch cost in the two samples. (a) & (c) respectively represent mean RT in the switch and task repetition condition for Sample 1 and Sample 2. (b) & (d) respectively represent individual differences related to switch cost for Sample 1 and Sample 2. Each circle represents a participant’s switch cost. The solid line indicates zero switch cost, and the dashed line represents the mean switch cost.

Mean RTs and accuracy data for all conditions (for both samples) are presented in Table 2 and Fig 3. Paired samples T-test for RT and accuracy showed that subjects consumed longer time [*t(31)* = 7.17; *p* < 0.001] and made more errors [*t(31)* = 3.35; *p* < 0.01] when responding to switching trials than repetition trials in Sample 1. The results derived from Sample 2 are similar to that from Sample 1: RT [*t(27)* = 5.09; *p* < 0.001] and Accuracy [*t(27)* = 3.92; *p* < 0.01]. These results indicated a significant switch cost in the two samples (Fig 3a and 3b). As shown in Fig 3c and 3d, there was a substantial amount of inter-individual variability in switch cost.

**Table 2:** Behavioral Results for Two Independent Samples.

	Sample 1		Sample 2	
	Switch	Repetition	Switch	Repetition
Mean RT (SD)	677.16 (125.25)	622.55 (133.67)	676.80 (196.59)	621.24 (164.33)
Accuracy (SD)	0.94 (0.04)	0.97 (0.03)	0.95(0.03)	0.97 (0.02)

RT = reaction time.

SD = standard deviation.

### Brain–behavior correlation data

Firstly, we calculated mean RTs and accuracy for each condition (Switching, Repetition); the calculation of RT excluded data from the first trial of each block, error trials, post-error trials and outlier trials (more than three standard deviations from the mean, calculated for each condition separately). Switch cost was determined by subtracting the mean RT of repetition trials from the switch trials.

**Fig 4:**
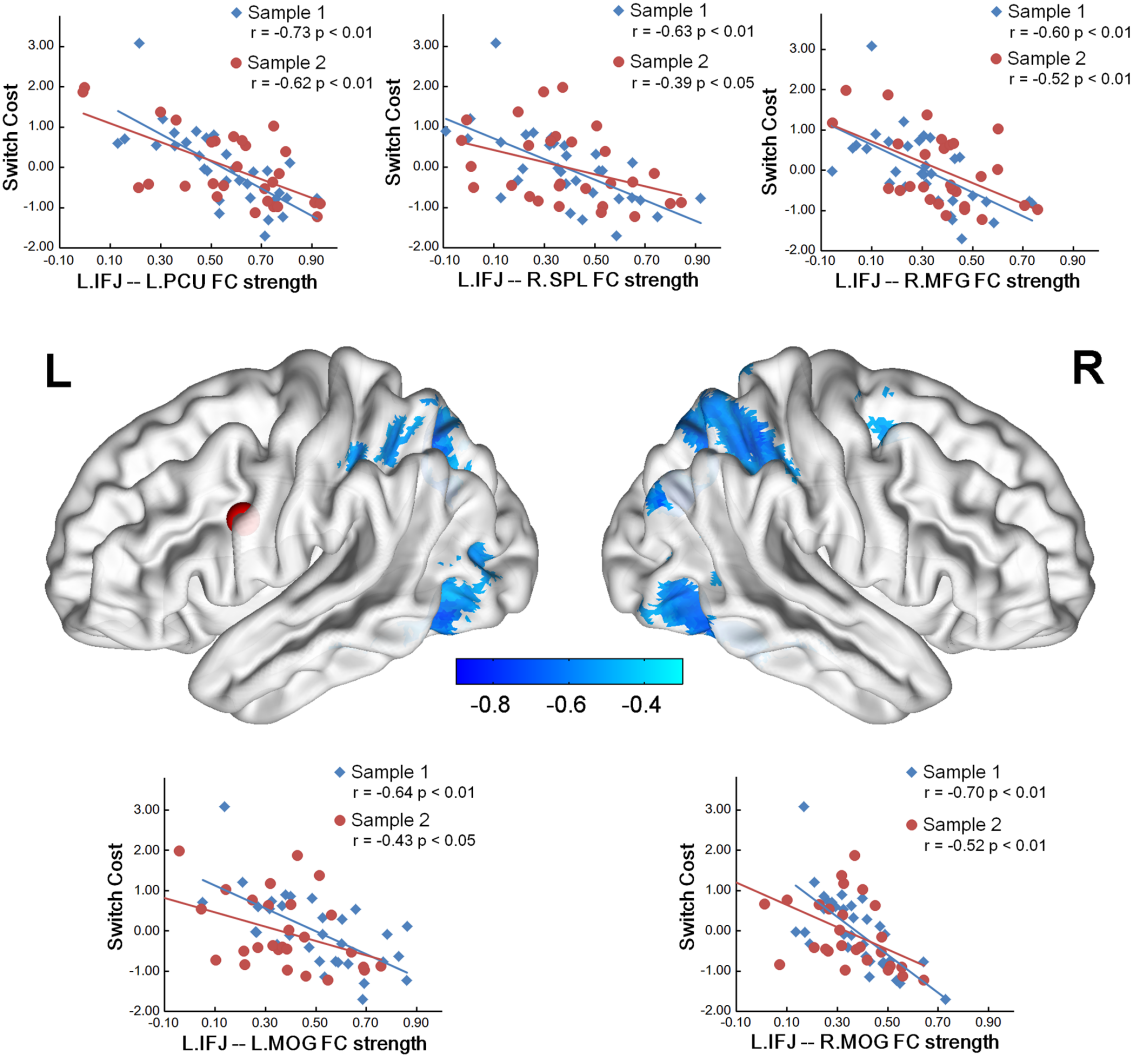
Switch cost relevant iFCs in the left IFJ. The red node represents the location of the left IFJ. Scatter plots with lines of best linear fit show the correlation between standard behavioral switch cost and iFC values in sample 1 and sample 2. Each dot represents data for a single subject. Note: L = left; R = right; FC = functional connectivity; IFJ = inferior frontal junction; PCU = precuneus; SPL = superior parietal lobule; MFG = middle frontal gyrus; MOG = middle occipital gyrus.

**Fig 5:**
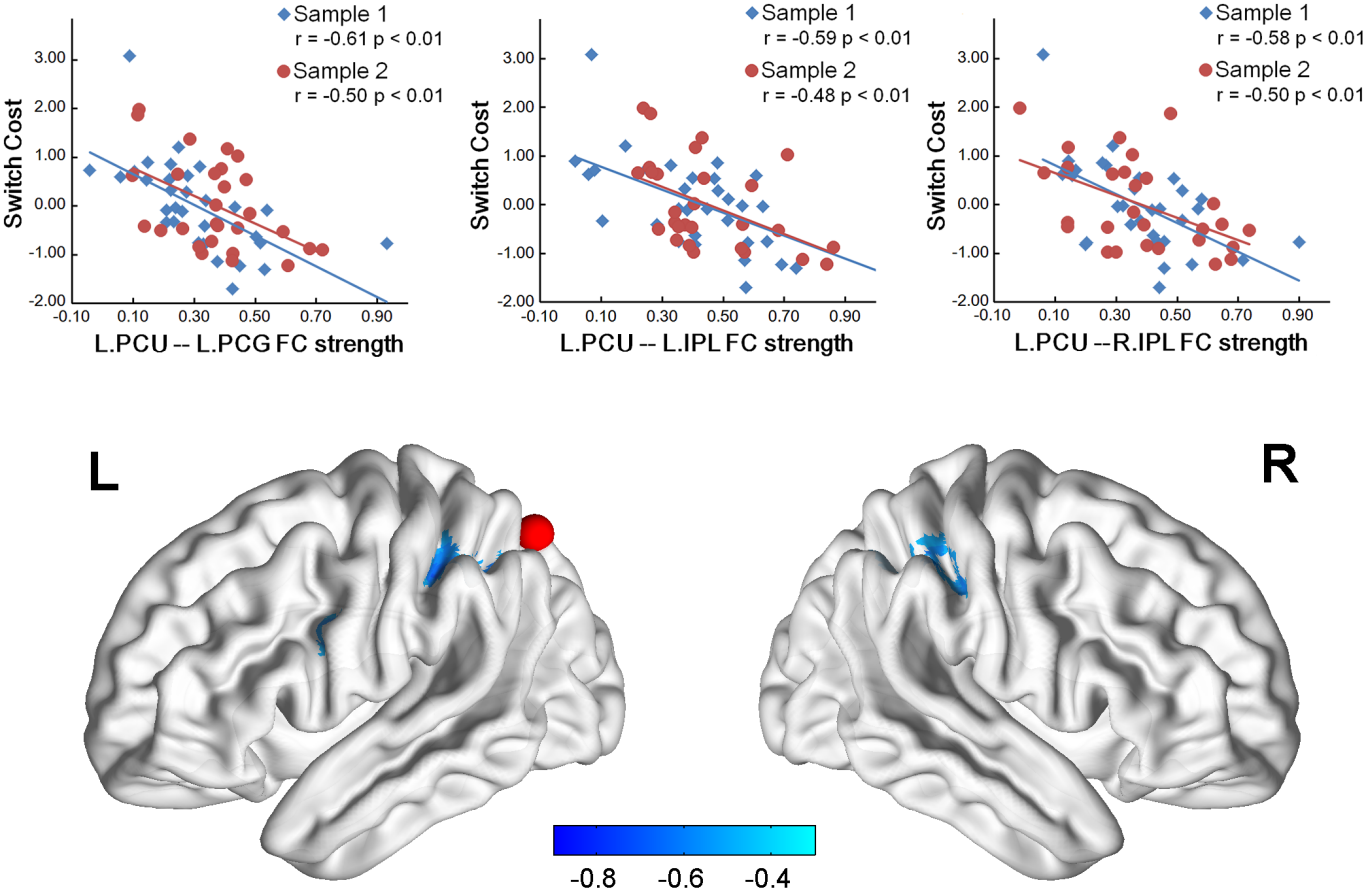
Switch cost relevant iFCs in the left precuneus. The red node represents the location of the left precuneus. Scatter plots with lines of best linear fit show the correlation between standard behavioral switch cost and iFC values in sample 1 and sample 2. Each dot represents data for a single subject. Note: L = left; R = right; FC = functional connectivity; PCU = precuneus; PCG = precentral gyrus; IPL = inferior parietal lobule.

By correlating the iFC strength of task-switching-related ROIs with switch cost, we found that multiple iFCs of the seeds, including left pre-SMA, bilateral inferior parietal lobule (IPL), the left IFJ and the left precuneus, negatively correlated with switch cost in Sample 1. After the test-validation procedure, the iFCs of the left IFJ and the left precuneus as seeds were reproducible in Sample 2 (Table 3). The iFCs between the left IFJ as seed and the left middle occipital gyrus (MOG, BAs 19 and 37, *r* = -0.64, *p* < 0.001), the right middle occipital gyrus (MOG, BAs 19 and 37, *r* = -0.70, *p* < 0.001), the right superior parietal lobule (SPL, BAs 7 and 40, *r* = -0.63, *p* < 0.001), the left precuneus (BAs 7 and 40, *r* = -0.73, *p* < 0.001), the right middle frontal gyrus (MFG, BA 6, *r* = -0.60, *p* < 0.001) were significantly detectable for predicting individual differences in switch cost (Fig 4). The iFCs between the left precuneus as seed and the left precentral gyrus (BA 6, *r* = -0.61, *p* < 0.001), the left IPL (BA 40, *r* = -0.59, *p* < 0.001), the right IPL (BA 40, *r* = -0.58, *p* < 0.001) significantly contributed to the prediction of switch cost (Fig 5). These correlation results remain significant when different strategies to correct head motion effects or global signal were conducted (Table 4). Furthermore, PPC showed significant iFC-switch cost correlation in the partial correlation mapping of L.IFJ. These regions (Table 5) included the right precuneus (BA 7, *r* = -0.44, *p* < 0.001), the left SPL (BA 7, *r* = -0.48, *p* < 0.001) and the right SPL (BA 7, *r* = -0.51, *p* < 0.001).

**Table 3:** Significant iFC-Switch Cost Correlation.

ROIs	Connected Regions	Peak Coordinates			Cluster size (voxels)	BA	Peak r-value	r-value in Sample2
		x	y	z				
*L*.*IFJ*	Left MOG	-45	-66	-9	393	37/19	-0.74	-0.43
	Right MOG	54	-66	-12	522	37/19	-0.77	-0.52
	Right SPL	33	-72	30	656	7/40	-0.77	-0.39
	Left PCU	-21	-63	48	363	7/40	-0.76	-0.62
	Right MFG	33	-3	66	84	6	-0.65	-0.52
*L*.*PCU1*	Left PCG	-45	0	27	57	6	-0.69	-0.50
	Left IPL	-36	-36	42	104	40	-0.71	-0.48
	Right IPL	42	-36	48	105	40	-0.65	-0.50

Abbreviations: IFJ = inferior frontal junction; MOG = middle occipital gyrus; SPL = superior parietal lobule; PCU = Precuneus; MFG = medial frontal gyrus; PCG = precentral gyrus; IPL = inferior parietal lobule.

**Table 4:** The r-values of Impacts of Different Preprocessing Strategies.

ROIs	Connected Regions	Motion Rigid-6	Motion Scrubbing	GSR	Present
*L*.*IFJ*	Left MOG	-0.62	-0.59	-0.47	-0.64
	Right MOG	-0.67	-0.70	-0.56	-0.70
	Right SPL	-0.47	-0.61	-0.47	-0.63
	Left PCU	-0.47	-0.62	-0.43	-0.73
	Right MFG	-0.45	-0.54	-0.40	-0.60
*L*.*PCU1*	Left PCG	-0.41	-0.51	-0.40	-0.61
	Left IPL	-0.50	-0.65	-0.53	-0.59
	Right IPL	-0.46	-0.50	-0.46	-0.58

Abbreviations: GSR = global signal regression; IFJ = inferior frontal junction; MOG = middle occipital gyrus; SPL = superior parietal lobule; PCU = Precuneus; MFG = medial frontal gyrus; PCG = precentral gyrus; IPL = inferior parietal lobule.

**Table 5:** Regions Showing Significant iFC-Switch Cost Correlation in the Partial Correlation Mapping of L.IFJ.

Connected regions	Peak Coordinates			Cluster size (voxels)	BA	Peak r-value
	x	y	z			
Right PCU	6	-63	33	109	7	-0.44
Right SPL	39	-48	57	147	7	-0.48
Left SPL	-30	-57	60	172	7	-0.51

Abbreviations: PCU = Precuneus; SPL = superior parietal lobule.

## Discussion

The present study investigated the relationship between the intrinsic functional connectivity (iFC) and individual differences in task-switching. We used a switching task to obtain the individuals’ switch cost, and examined the correlation between the iFC maps and switch cost. We observed that lower switch cost was related to higher iFC between the left IFJ and bilateral PPC, between left precuneus and bilateral IPL, between left IFJ and right MFG, between left IFJ and bilateral MOG. These findings were reproducible across two independent samples and highly reliable across different preprocessing as well as different head motion correcting strategies. Furthermore, PPC showed significant iFC-switch cost correlation in the partial correlation mapping of L.IFJ. The regions in our iFC results, including IFJ, IPL, SPL and precuneus were all Included in the 11 switch-related meta-regions. These connectivity profiles represent an intrinsic functional architecture of task-switching where the left IFJ plays a hub role in this brain-behavior association.

Many fMRI studies have been conducted to investigate the specific function to the activated brain regions in task-switching. A large body of evidence has demonstrated a role for PPC in representing a task-set [40, 41]. Furthermore, the activation in PPC, including superior and inferior PPC (BA 7, 40), is consistently detected during re-activating or representing task-sets when individuals perform a switching task [42–44]. Considering the present results, we speculate that the connection between the left precuneus (in superior PPC) and bilateral IPL (in inferior PPC) may contribute to the task-sets representing processing in task-switching. In a recent study, De Baene et al. (2012) used an adaptation approach to differentiate the brain areas selectively representing the what and the how components of cognitive control in task preparation, and found that superior PPC preferentially represented task goal, namely the “what” component; whereas inferior PPC preferentially represented S-R mapping, namely the “how” component [45]. It showed that superior and inferior PPC were co-activated in task-switching to represent task-sets including the goal and the S-R mapping. Our finding expands the previous findings and indicated the intrinsic functional architecture of the representation for task-sets.

A lot of task-switching studies have also argued the general role of left IFJ in updating a task-set [45–47]. The IFJ is located at the junction of the inferior frontal sulcus and the inferior precentral sulcus, which is a posterior location in the lateral prefrontal cortex. Brass et al. (2005) summarized past literatures and emphasized the pivotal role of left IFJ in promoting the interaction of information [11]. Our finding that the strength of these left IFJ-seeded iFCs was negatively correlated with switch cost in task performance also highlighted the critical role that the left IFJ plays in supporting the intrinsic functional architecture of task-switching. In a recent fMRI study of task switch, researchers identified that the left IFJ and left PPC contribute to core cognitive processes, that is, representing and updating task-sets, generic to task-switching [46]. Furthermore, the results of diffusion tensor imaging (DTI) tractography studies showed that IFJ and PPC are not only anatomically connected via the superior longitudinal fasciculus (SLF) [48], but this strength of anatomical connectivity (assessed via DTI metric of fractional anisotropy) also can predict switch cost in young and older adults [49]. The anatomical connectivity suggested that there should be functional interactions of information between IFJ and PPC. The present results go one step further by suggesting that the strength of iFC between the left IFJ and bilateral PPC could also predict switch cost and indicating the intrinsic functional basis of the information interaction between them.

The iFC strength between the left IFJ and the right MFG negatively affected the behavior performance of a switching task. The present coordinate of right MFG showed that this region was a rostral portion of dorsal premotor cortex (pre-PMd). Rushworth and colleagues (2002) conducted a repetitive transcranial magnetic stimulation (rTMS) study and found that a disruptive effect of pre-PMd rTMS is seen regardless of whether or not subjects are switching set; whereas the disruptive effect of pre-SMA rTMS is seen only when subjects are switching set, which suggested that pre-PMd’s subordinate role in selecting between individual responses [50]. In this sense, the left IFJ seems to play a top-down controller in selecting the corresponding response output. Both the left IFJ and early visual cortices was found in the current study with the iFC between them associated with the task-switching cost. Considering the relatively lower level roles of visual cortices, the iFC seems to reflect intrinsic functional architecture of selecting the relevant visual input. Our finding demonstrated the intrinsic functional architecture to support this possible information flow. Even though this is just one possibility, a recent fMRI study showed similar results. Stelzel et al. (2011) found increased connectivity between the left IFJ and motor regions, between left IFJ and MOG in a switching task, showing the switching-related interaction of these regions [51].

Furthermore, it is intriguing that the partial iFC (controlling for the signal from another ten switch-related regions) between L.IFJ and PPC still significantly contributed to the switch cost. This reproducible result highlights the fundamental role of iFC between L.IFJ and PPC in intrinsic functional architecture of task-switching. Switching between tasks requires individuals to flexibly update the new task settings. These augmented functional interactions in subjects with faster switching may facilitate close cooperation between the system used for updating task-sets (left IFJ) and that used for representation of task-sets (PPC). However, these conclusions are speculative and remain to be confirmed through combination of both task-related fMRI and rfMRI studies.

In the present findings, numerous brain regions are functionally connected to the left IFJ, regarding the task-switching performance, which could have at least two important implications for understanding the neural basis of task-switching. First, it shows that updating a task-set, which involves left IFJ, is a critical process in task-switching. Task-sets updating reflect the active control conducted by individual to fulfill the task demands, which is one source of switch costs [1]. Second, these iFC profiles support the speculation that the left IFJ provide a kind of hub to integrate task switch-related information. This viewpoint had already been proposed in many neuroimaging studies of task-switching [45, 46, 52]. Our results thus show the intrinsic functional basis for this viewpoint. The cognitive functions are attributable to the dynamic interactions of distributed brain areas operating in large-scale networks [53]. Resting-state fMRI data provide substantial advances in understanding brain functional organization systematically instead of identifying a special function for one region. Researchers had proposed one reasonable hypothesis based on the Hebbian mechanism [54] to describe the nature of correlated spontaneous BOLD fluctuations. That is, the correlated spontaneous BOLD fluctuations may, at least in part, reflect a long-standing history of co-activation [12]. On the other hand, iFC may aid to keep functional systems in an active state, helping to improve performance [55]. Here, it is reasonable to speculate that the strength of these iFCs most likely reflects the degree of functional consistency in task-switching. Meanwhile, the closer relationship between these regions contributes to the better performance in task-switching. However, because it is lack of direct evidence, how the information flow between these brain regions for online task-switching is still speculative and need to be further explored in future. A large body of resting-state studies has now built a substantial empirical basis for the claim that brain regions that are coactive during a task tend to have correlated low-frequency rfMRI signal [56–58]. Thus, our exploration results provide the hypothesis for further studies to identify how these regions cooperate to achieve a special process in task-switching.

## Conclusions

In summary, we mapped out the functional connectivity pattern at rest that was related to switch cost. Specifically, stronger couplings between left IFJ and bilateral PPC, between left precuneus and bilateral IPL, between left IFJ and right MFG, between left IFJ and bilateral MOG, were associated with better behavior performance in task-switching. These results not only revealed the neural basis of individual difference for task-switching, but also show the intrinsic functional architecture of task-switching. Our data indicate the critical role of the left IFJ in the switch-related intrinsic functional architecture. The present work provides a novel perspective for understanding the neural basis of task-switching, and also promotes further studies to directly identify the functional roles of these intrinsic profiles.
